# Admission of Women to the Study of Veterinary Medicine

**Published:** 1896-09

**Authors:** 


Admission of Women to the Study of Veterinary Medicine. Dr. Swetlin has written an article for the Vienna Chamber of Physicians concerning the study to be pursued by women, and addressed to the Chamber of Deputies. It contains the following remarkable passage: "Let the Chamber take cognizance of the fact that the Chamber of Physicians, with full knowledge of the difficulties and demands of the calling as physician, declares women to be less adapted to carry out the practice of human medicine. May the Chamber of Deputies, in consideration of the objective point of view which the Vienna Chamber of Physicians has taken, never consent to the admission of women to the study of medicine alone, but open the doors of all the faculties—pharmacy, veterinary medicine, technology, agriculture, and business to the graduates of the girls' gymnasiums, and, finally, to make admission to the high schools dependent upon finishing a course in the girls' gymnasium which shall correspond exactly to that of the boys, and presenting from the same a certificate of maturity."—*Thierärztliches Centralblatt*, March 1, 1896.

